# Psychological symptoms and salivary inflammatory biomarkers in patients with dentofacial deformities: a case–control study

**DOI:** 10.1038/s41598-021-90721-6

**Published:** 2021-05-26

**Authors:** Maria C. C. Volkweis, Gabriela W. Neculqueo, Raquel D. S. Freitas, Ana P. A. Dagnino, Guilherme G. Fritscher, Tatiana Q. Irigaray, Maria M. Campos

**Affiliations:** 1 Programa de Pós-graduação em Odontologia, Escola de Ciências da Saúde e da Vida, Pontifícia Universidade Católica do Rio Grande do Sul, Avenida Ipiranga, 6681, Partenon, Porto Alegre, RS 90619-900 Brazil; 2 Programa de Pós-graduação em Medicina e Ciências da Saúde, Escola de Medicina, Pontifícia Universidade Católica do Rio Grande do Sul, Porto Alegre, RS Brazil; 3 Centro de Pesquisa em Toxicologia e Farmacologia, Escola de Ciências da Saúde e da Vida, Pontifícia Universidade Católica do Rio Grande do Sul, Porto Alegre, RS Brazil; 4 Ambulatório de Cirurgia Oral, Escola de Ciências da Saúde e da Vida, Pontifícia Universidade Católica do Rio Grande do Sul, Porto Alegre, RS Brazil; 5 Programa de Pós-graduação em Psicologia, Escola de Ciências da Saúde e da Vida, Pontifícia Universidade Católica do Rio Grande do Sul, Porto Alegre, RS Brazil

**Keywords:** Dentistry, Quality of life

## Abstract

Individuals with dentofacial deformities often display a low quality of life (QoL) through biological mechanisms that remain unraveled. In this case–control study, the salivary levels of cytokines, glutamate, and kynurenine metabolites were assessed in patients undergoing orthognathic surgery (OS), while correlating these parameters with QoL and psychological symptoms. Thirty-six patients were enrolled in control (under orthodontic treatment) and test (undergoing OS) groups, matched by age and sex. The QoL was assessed through the World Health Organization Quality of Life BREF (WHOQOL-BREF) and the Orthognathic Quality of Life Questionnaire (OQLQ). The psychological symptoms were evaluated by the Satisfaction with Life Scale, the Rosenberg Self-Esteem Scale (RSES), and the Depression, Anxiety, and Stress Scale-21 (DASS-21). The salivary levels of IL-1β, IL-6, IL-10, glutamate, and kynurenine metabolites were evaluated. The OQLQ demonstrated increased QoL scores in the test group, regarding social aspects, facial esthetics, and function domains, without significant differences in respect to the other surveys. These patients displayed higher IL-1β and glutamate levels; conversely, the kynurenine metabolites were unaltered. The glutamate levels positively correlated with the OQLQ function scores. The data brings novel evidence about the psychobiological features of patients with dentofacial deformities, showing salivary variations of inflammatory biomarkers in these individuals.

## Introduction

Individuals with severe dentofacial deformities require orthognathic surgery (OS), in addition to orthodontic treatment. In most cases, the affected patients need to undergo a long-term preparation before surgery. Recent evidence has demonstrated that psychological and esthetic factors have a considerable impact on the QoL of the affected individuals^1^. A previous study has demonstrated that patients with dentofacial deformities who presented depression had a poorer QoL, relating to vitality, social aspects, and mental health, as evaluated one year before the surgery^2^. Similarly, a study carried out with 140 patients, before and after OS, showed that the correction of orofacial deformities led to QoL improvements, and depression was inferior in the individuals with high preoperative depression scores^3^. Of note, poorer QoL scores were registered for the subjects living with dentofacial abnormalities when in comparison with the control individuals, or with the patients previously submitted to OS^4^. Extending this evidence, a study by Sun et al. demonstrated that patients with dentofacial deformities presented a poorer QoL, mainly related to the functional and psychological dimensions^5^. However, there is scarce literature data about the potential biological mechanisms underlying the psychosocial aspects of the patients with dentofacial deformities that require surgical correction. To the best of the authors’ knowledge, only one study has correlated the polymorphisms of the dopaminergic system-related ANKK1 gene, against the perception of the QoL in the patients who underwent OS^6^, indicating the need for further studies on this topic.

During the last decades, compelling evidence has suggested an intimate relationship between inflammation and the social processes^7^. In this regard, inflammatory mediators, mainly cytokines, trigger the activation of the kynurenine pathway via the stimulation of indoleamine 2,3-deoxygenase (IDO-1), accounting for psychological disorders, such as bipolar disorders^8^. Moreover, chronic stress has been associated with an increased production of inflammatory cytokines and the stimulation of the kynurenine pathway, mediating the tonic activity of the sympathetic nervous system in depression^9^. As reviewed by Arnone et al., kynurenine metabolites, namely the kynurenic and quinolinic acids, are likely involved in a depressed mood^10^. Besides, there is a clear link between the activation of the inflammatory pathways and a massive release of glutamate from the glial cells, as a core of mood disorders^11^. Interestingly, it has been proposed that inflammation might present a protective role in helping to process the social defeats^12,13^.

In light of the abovementioned data, it is tempting to presume that individuals with dentofacial deformities who require surgery present differences in the levels of inflammatory mediators when related to their social context, poor QoL, and depression development. Thus, the present study has aimed to analyze the salivary levels of cytokines, glutamate, and kynurenine metabolites in patients with dentofacial deformities undergoing OS, correlating these findings with the psychosocial aspects of this group of individuals.

## Results

### Demographic data

The age of participants in this study ranged from 15 to 48 years. The mean age of the individuals in the control and test groups was 24.6 ± 7.7 and 25.2 ± 6.8 years, respectively (*t* = 0.2463). The percentage of females was about 62% in either group. Besides, most of the participants from both of the groups had completed high school. Irrespective of the group, a higher percentage of individuals were single, Caucasians, cited regular physical activities, good self-perceived oral health, and the absence of sleeping problems. Monthly incomes and the number of people cohabitating were similar for the participants that were allocated into either group. There were no significant differences when comparing the body mass index (BMI/BMI z-score) between the control (23.9 ± 3.9) and the test groups (23.5 ± 3.8) (*t* = 0.3612). For the test group, the most common type of deformity was prognathism. This data set is summarized in Table 1. There were no differences between the control and the test groups when considering religion (mostly catholic), domestically-owned animals (mainly dogs), or the main occupation (student was the main occupation) (Supplementary Table S1). The main complaint that led the patients to seek an orthodontic treatment (control group) was esthetics (8/19), whereas 6 and 5 individuals in this group mentioned function or esthetic/function, respectively. The patients needing orthodontic-orthognathic treatment (test group) reported esthetics plus function as the main complaint (10/17), with 3 and 4 subjects reporting esthetics or function only, respectively (*Chi-square* = 4.1; *p*-value = 0.0428; results not shown).

**Table 1:** Data regarding the socio-demographic aspects, habits, self-perceived oral health, and type of deformity.

	Groups		*p*-value
	Control	Test	
N	19	17	
**Sex**			
Male	9	8	> 0.9999
Female	10	9	
Age range (y)	15–48	15–41	
Mean ± SD age (y)	24.6 ± 7.7	25.2 ± 6.8	0.8070
Mean ± SD height (m)	1.65 ± 0.1	1.68 ± 0.1	0.3752
Mean ± SD weight (kg)	67.1 ± 14.6	70.4 ± 19.4	0.5654
BMI	23.9 ± 3.9	23.5 ± 3.9	0.7202
**Educational level**			
Middle school	2	2	0.7991
High school	13	12	
College	4	3	
**Ethnicity**			
African American	–	2	0.2159
Caucasian	19	15	
**Monthly household income (BR$)**			
< 1000	–	1	0.1553
1000–3000	6	7	
3000–5000	6	6	
> 5000	7	3	
**Marital status**			
Single	17	15	> 0.9999
Married or in a relationship	2	2	
**Residing**			
Alone	3	4	0.1136
1 person	4	5	
2 people	5	7	
3 or more	7	1	
**Sleep disorders**			
Yes	4	1	0.3353
No	13	16	
**Physical activity**			
Sedentary	6	10	0.1787
Exercise practice	13	7	
**Self-perceived oral health**			
Good	15	13	0.8656
Fair	3	4	
Poor	1	–	
**Type of deformity**			
Prognathism	–	13	–
Retrognathism	–	4	

*n*, participants in each group; *y*, years; *m*, meter; *kg*, kilograms; *BMI*, body mass index; *BRL*, Brazil’s currency. Statistical analysis was performed by Unpaired Student’s t-test or chi-square test for trend.

### Assessment of life quality parameters

**Figure 1:**
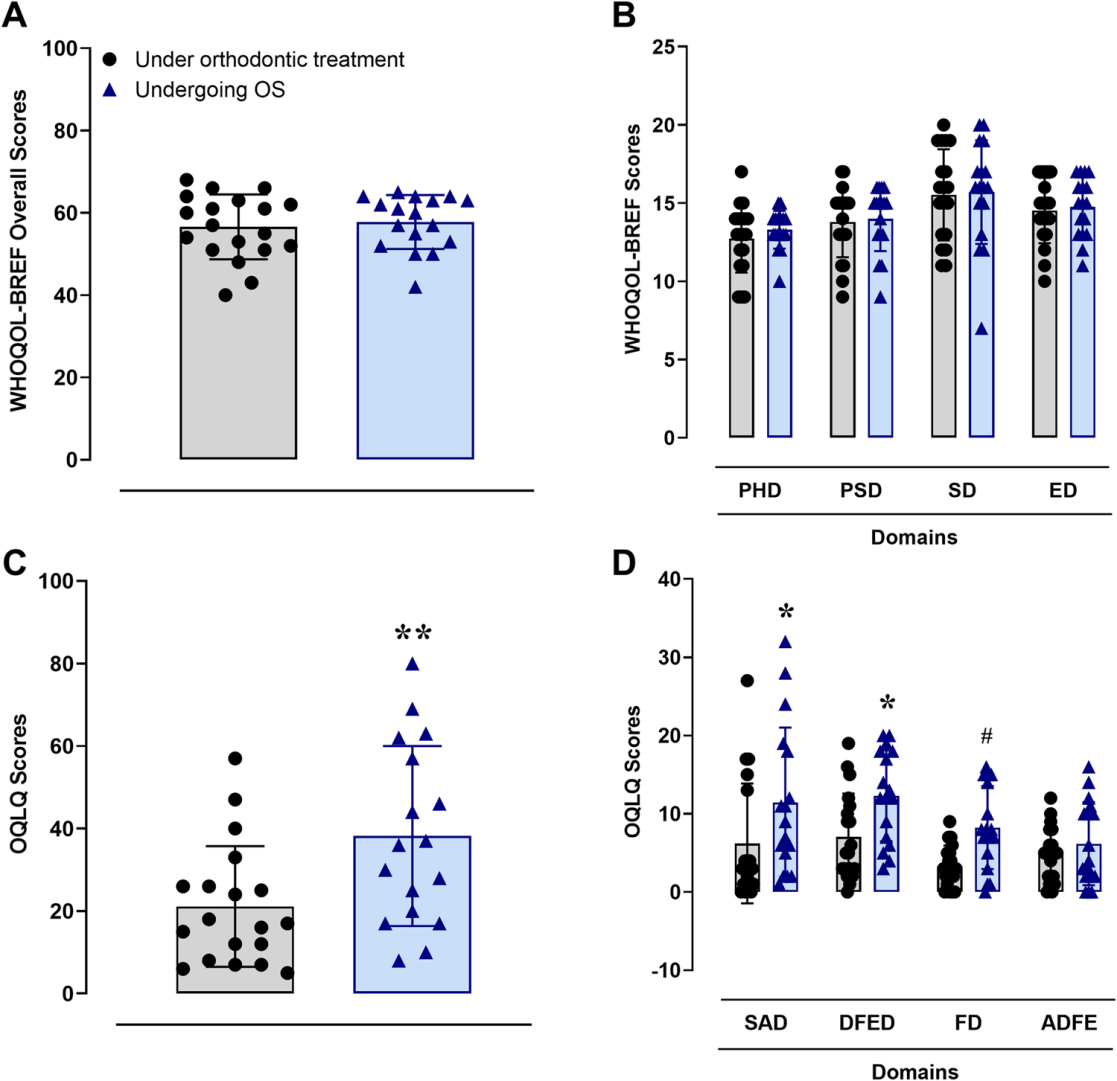
World Health Organization QoL Scale (WHOQOL-BREF) total scores (**A**), WHOQOL-BREF domain scores (**B**), Orthognathic QoL Questionnaire (OQLQ) total scores (**C**), and OQLQ domain scores (**D**), for the control (n = 19) and test (n = 17) groups. For the WHOQOL instruments, the Unpaired Student’s t-test (**A**), and Two-Way ANOVA (**B**) were performed. As for the OQLQ tests, the Unpaired Student’s t-test (**C**), and Two-Way ANOVA followed by Sidak’s test (**D**) were performed, where the test group was significantly different from the control group (*p < 0.05; **p < 0.01; ^#^*p* = 0.054). Each column represents the mean ± SD, and the N of each group is depicted in the scatter dot plots. The WHOQOL domains were the physical health domain (PHD), the psychological domain (PSD), the social domain (SD) and the environment domain (ED). The OQLQ domains were the social aspects domain (SAD), the dentofacial esthetics domain (DFED), the function domain (FD), and the awareness of dentofacial esthetics (ADFE).

The WHOQOL-BREF instrument was used to evaluate the QoL of the participants. There were no significant differences concerning questions 1 (overall QoL) and 2 (general health) between the control and test groups. The mean ± SD for question 1 was 4.2 ± 0.6 (control) and 3.8 ± 0.39 (test) (*t* = 1.947; *p*-value = 0.06). For question 2, the mean scores (accompanied by SD) were 3.9 ± 0.8 and 3.8 ± 0.4, for the control and test groups, respectively (*t* = 0.7595; *p*-value = 0.4581). Furthermore, the sum of the QoL scores did not significantly differ between the control and the test groups (Fig. 1A; *t* = 0.4863). The same questionnaire did not reveal any significant difference between the groups, according to the separate evaluation of the physical, psychological, social, and environment domains (Fig. 1B; *F* = 0.0513). The patients that were allocated to the test group presented significantly higher scores in the OQLQ when in relation to the control group (Fig. 1C; *t* = 2.786) The analyses of the OQLQ domains further confirmed this indication of poorer QoL for the orthognathic patients. The scores for social aspects, facial esthetics, and functional domains were significantly higher for the test group, whereas the dimension of awareness of facial deformity did not show any difference (Fig. 1D; *F* = 0.8965).

### Psychological symptoms

**Figure 2:**
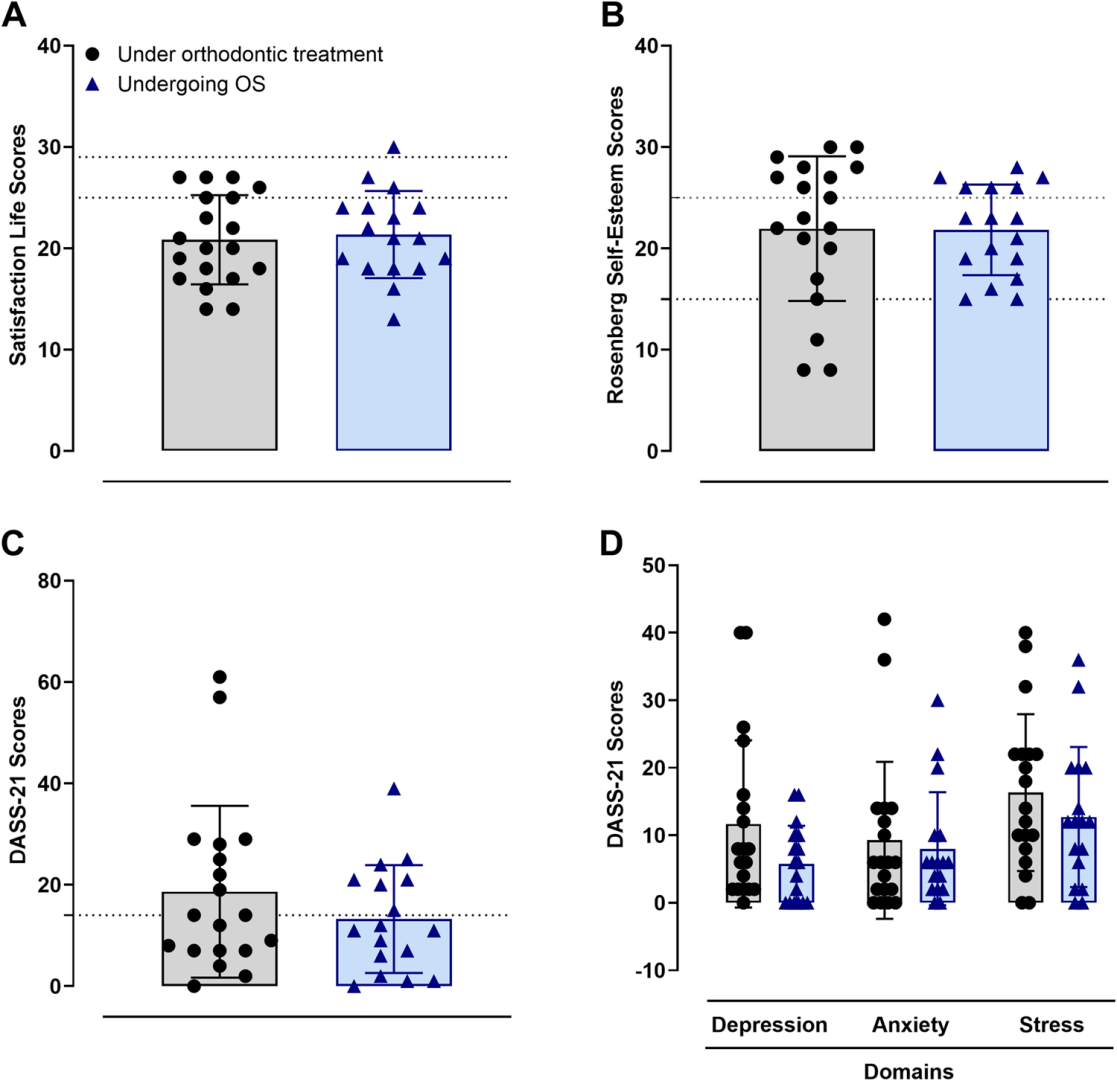
Satisfaction with Life Score (**A**), Rosenberg Self-Esteem Score (**B**), Depression Anxiety Stress Scale 21 (DASS-21) total scores (**C**), and DASS-21 domain scores (**D**): depression, anxiety, and stress in the control (n = 19) and test (n = 17) groups. For the Satisfaction with Life Score, the Rosenberg Self-Esteem Score, and the DASS-21 total score, the Unpaired Student’s t-test was performed. As for the DASS-21 domain scores, Two-Way ANOVA was performed. No significant differences were observed between the experimental groups (*p* > 0.05). Each column represents the mean ± SD, and the N of each group is depicted in the scatter dot plots.

The patients next responded to the Satisfaction with Life (Fig. 2A) and the RSES (Fig. 2B) questionnaires. There were no significant differences when comparing the scores of life satisfaction and self-esteem (*t* = 0.3515 and *t* = 0.0615, respectively), while most individuals in both of the groups scored adequate levels of self-esteem (Fig. 2B). The participants of the study were also evaluated by responding to the DASS-21 instrument. Neither the total scores (Fig. 2C; *t* = 1.129), nor the depression, anxiety, or stress domains (Fig. 2D; *F* = 0.4524) were significantly different between the control and test groups. Further analyses of the DASS-21 scale results did not show any difference in the frequencies of the severity of depression, anxiety, or stress symptoms when these domains were rated from normal to extremely severe in both of the groups (Supplementary Table S2).

### Biochemical evaluation of salivary biomarkers

**Figure 3:**
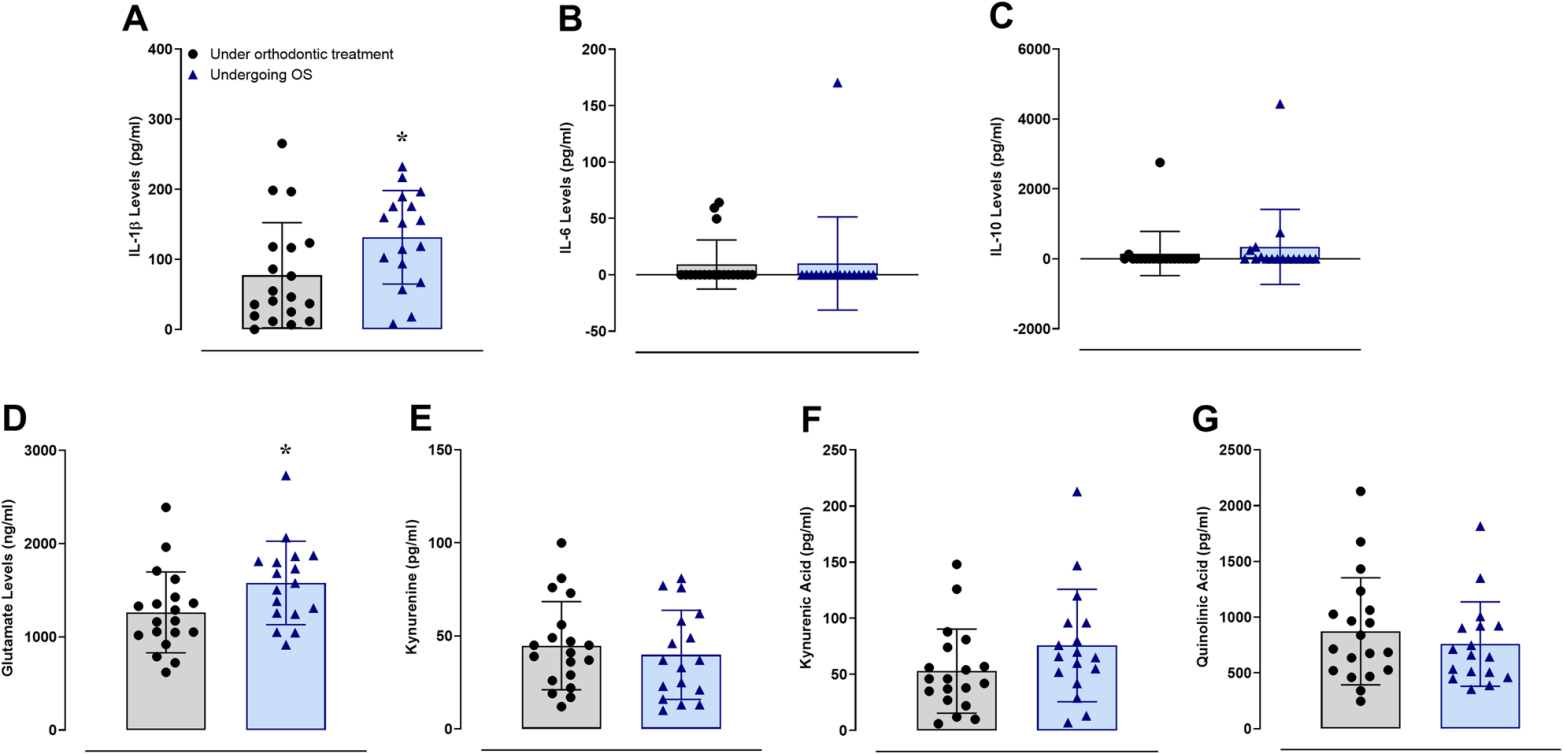
The salivary levels of IL-1β (**A**), IL-6 (**B**), IL-10 (**C**), glutamate (**D**), kynurenine (**E**), kynurenic acid (**F**), and quinolinic acid (**G**) in the control (n = 19) and test (n = 17) groups, according to the assessment by the DuoSet ELISA assay and UHPLC-MS. The Unpaired Student’s t-test was performed for all of the inflammatory markers, and the test group was significantly different from the control group (A and D) (*p < 0.05). Each column represents the mean ± SD, and the N of each group is depicted in the scatter dot plots.

The salivary levels of the pro-inflammatory cytokine IL-1β were significantly higher in the test group than in the control individuals (Fig. 3A; *t* = 2.276). Alternatively, the levels of the pro-inflammatory cytokine IL-6 (Fig. 3B; *t* = 0.0842) or the anti-inflammatory cytokine IL-10 (Fig. 3C; *t* = 0.6604) were not significantly different. Curiously, the salivary glutamate contents were significantly increased in the test group when in relation to the control group (Fig. 3D; *t* = 2.149). On the other hand, the components of the tryptophan-kynurenine pathway, namely kynurenine (Fig. 3E; *t* = 0.6186), kynurenic acid (Fig. 3F; *t* = 1.558), or quinolinic acid (Fig. 3G; *t* = 0.7900), presented similar salivary amounts between both of the groups.

### Correlation analysis

To correlate the psychosocial determinants and the biochemical results, Pearson’s correlation analysis was carried out. A positive correlation was observed between the salivary glutamate contents and the function domain scores of the OQLQ instrument (*r* = 0.61; *p* = 0.0095), according to the evaluation of the test group. The elevated salivary levels of IL-1β did not show any correlation with the OQLQ scores (*r* = − 0.24; *p* = 0.35), or with the social aspects (*r* = − 0.28; *p* = 0.27), the dentofacial esthetics (*r* = − 29; *p* = 0.26), and the function domains (*r* = 0.04; *p* = 0.86). No significant correlation was observed between the glutamate levels and the OQLQ scores (*r* = 0.31; *p* = 0.22), or the social aspects (*r* = 0.09; *p* = 0.74) and the dentofacial esthetics (*r* = 0.27; *p* = 0.29) domains. An overall analysis did not show a significant correlation between age, and the IL-1β (*r* = 0.056; *p* = 0.74) or the glutamate (*r* = − 0.09; *p* = 0.58) levels. Additionally, no significant correlation was found between the BMI, and IL-1β (*r* = 0.1879; *p* = 0.27) or glutamate (*r* = − 0.13; *p* = 0.44) contents.

## Discussion

It has been demonstrated that psychosocial and esthetic factors have a great impact on the QoL of individuals with dentofacial deformities that require corrective surgery^3^, despite the lack of studies investigating the biological basis of these alterations. Noteworthy, the recent literature data has suggested a close relationship between inflammation and the social processes^12,13^, which might well be implicated in the psychological features of the patients undergoing OS. Therefore, the present study has aimed to evaluate the salivary levels of inflammatory biomarkers in the patients with dentofacial deformities undergoing OS, whilst correlating these findings with the QoL and psychological parameters. The null hypothesis was that the patients in preparation for OS did not exhibit differences regarding the salivary levels of cytokines, glutamate, or kynurenine metabolites when in relation to the control patients without surgical needs.

In this study, the authors considered the participants from the control group that were under an orthodontic treatment, without the recommendation for OS. This was because the patients in preparation for an orthognathic correction (considered here as the test group) were using an orthodontic device. This criterion was fundamental because orthodontic treatment has been previously correlated with salivary changes of inflammatory mediators, such as prostaglandin E2 and IL-1β^14^. Furthermore, the control group participants' sociodemographic features were similar to those seen in the test individuals undergoing OS, showing homogeneity of the study sample, regarding age, sex, BMI, educational level, ethnicity, and socioeconomic aspects, as well as the self-reported sleep quality, oral health status, and physical activity habits. Most patients who were included in the test group referred to both esthetics and functional reasons for seeking OS, whereas the control patients indicated esthetics or function individually, as the primary motivation for the orthodontic correction treatments. In this study, 13 of 17 patients presented prognathism, concerning the dentofacial deformity type, which corresponded to 76% of the test group. This data corroborates with a previous publication that showed a higher percentage of prognathic patients (78%), who sought orthognathic treatments in a Brazilian oral surgery center^15^.

A case–control study that was conducted by Lee et al. evaluated the general QoL of 76 patients undergoing OS when compared with control subjects, by using the 36-item Short-Form Health Survey (SF-36). The authors did not observe any significant difference between the control and the OS groups^16^. In this present study, the evaluation of patients by the WHOQOL-BREF instrument did not reveal any difference between the control and test groups, even when considering the separated dimensions of this questionnaire, such as the physical, psychological, social, or environmental aspects. Even so, the analysis of question 1 of this survey revealed a trend that differentiated the control from the test group (*p*-value = 0.06), indicating a slight difference in the overall QoL perception, with higher scores in the control group. Two additional studies employed the WHOQOL-BREF instrument to assess the QoL of patients before and after OS and they showed a similar range of QoL scores in the preoperative phase^3,6^.

The current results have demonstrated that the test group participants displayed higher scores in the OQLQ instrument when compared to the control individuals. As described previously, for this questionnaire, the higher scores indicated an impaired orthognathic QoL^16^. An additional assessment of the different domains of OQLQ revealed that the patients in the test group presented less favorable scores for the social aspects, dentofacial esthetics, or function domains, without any difference in the awareness of dentofacial esthetics. The results have agreed with previous data indicating that the patients undergoing OS display overall and specific OQLQ scores higher than those that were observed in the control group^16^. Still, this previous publication also detected differences in connection with the awareness of dentofacial esthetics when comparing the control and case groups. In our study, the control group was composed of patients undergoing an orthodontic treatment, while in the study by Lee et al.^16^, the control group was formed by patients who presented an asymptomatic third molar, which can help to explain this discrepancy. By extending our results somewhat, the evaluation of the oral health-related QoL, through the Oral Health Impact Profile (OHIP-J54), showed poorer scores in the patients undergoing OS when compared with the control individuals, with differences in the functional, physical, and social aspects^17^.

To gain further insights into the psychological factors of patients with dentofacial deformities, we decided to assess the levels of life satisfaction and self-esteem of the study’s population. Most of the subjects, in both of the control and test groups, presented life satisfaction scores, ranging from moderately unsatisfied (15–19) to moderately satisfied (20–24), with no significant differences between the groups^18^. Of interest, Eriksen et al.^19^ described a significant gain in the life satisfaction of patients, according to the assessment that occurred 10 to 15 years after the OS. It is possible to infer that orthodontic or orthodontic-orthognathic patients will present an improvement in the life satisfaction levels after completing their treatments. For the self-esteem scale in this work, the mean values for the control and test groups were approximately 22, which are considered adequate scores for the RSES^20^. Similarly, Alanko et al. reported values close to 23 when using the same instrument in a similar time-point as in this study^21^. Conversely, the prognathic patients showed lower pre- and post-surgical self-esteem scores when compared with the control patients in a study that was carried out by Agırnaslıgıl et al.^22^. This divergent result cannot be truly explained, but cultural differences might influence the levels of self-esteem and the impacts of dentofacial deformities regarding well-being and social acceptance. A Brazilian study has also detected significant differences between the control and experimental (pre-surgery) patients with reference to the Rosenberg Self-Esteem Scale (RSES) results^23^, contrasting somewhat to the proposition of cultural differences.

Despite the ideal self-esteem levels that were observed in both of the groups that were evaluated herein, the participants showed moderate life satisfaction levels. When viewing these results and the significant data that was obtained from the OQLQ, the scores of depression, anxiety, and stress symptoms in the study population were investigated. Worthy of attention, to the best of the authors' knowledge, this is the first study that has used the DASS-21 instrument to evaluate patients with dentofacial deformities. No significant differences between the control and test groups were observed, nor were overall differences noticed for the individual analyses of depression, anxiety, and stress symptoms. Even the frequency of subjects that were classified with normal to extremely severe scores of depression, anxiety, or stress, did not differ in either group. Most patients presented normal levels for the three evaluated dimensions, regardless of the group. Supporting the current data, Frejman et al. did not find any difference in the depression scores of the control and pre-OS patients when using the General Hospital Depression Scale^23^. Furthermore, the evaluation of psychological determinants in a group of 99 patients undergoing OS showed that most patients exhibited none to minor levels of anxiety (76 individuals), or depression (83 individuals), according to the Beck Anxiety Inventory (BAI), or the Patient Health Questionnaire-9 (PHQ-9), respectively^24^. Otherwise, the Symptom Checklist-90 (SCL-90) application revealed significant differences between the patients with dentofacial deformities and the controls regarding depression, but not anxiety^25^.

Studies investigating the biological pathways that are implicated in an altered QoL and psychosocial determinants in individuals with facial skeletal deformities are still lacking. In this work, the salivary levels of the inflammatory biomarkers were evaluated in the patients undergoing OS, in comparison with the control individuals that were under the orthodontic treatment. Saliva has been a low-cost and safe alternative to assess the inflammatory biomarker levels, having a good correlation with the serum results^26–28^. We opted for using stimulated saliva, by considering the greater inter-individual variations regarding the collecting volumes and the protein contents in the unstimulated saliva, as described beforehand^29^. Firstly, the levels of two pro-inflammatory cytokines were analyzed, namely IL-6 and IL-1β. While the IL-6 salivary levels were undetectable in most of the patients in both of the groups, the contents of IL-1β were detected in either the control or the test groups. Of particular interest, the IL-1β levels were significantly higher (+ 70%) in the test group with dentofacial deformities. It was previously demonstrated that the control patients displayed detectable salivary levels of IL-1β, and the orthodontic forces might drive an upregulation of this cytokine^14,30^, corroborating with the present investigation. The elevated IL-1β salivary levels in the test group might be attributed to the social context, or to the oral function disabilities that were observed in these patients. An interesting study revealed enhanced salivary levels of IL-1β in the individuals undergoing an academic exam, confirming the relevance of this cytokine in social stress conditions^31^. Similarly, another type of stressful condition (i.e., public speaking) has also led to increased salivary IL-1β contents, as a biomarker of adverse social situations^32^. No correlations between the IL-1β levels and the OQLQ scores were observed, even when considering the separate domains of the social aspects, the dentofacial esthetics, the function, and the awareness of dentofacial esthetics. Nonetheless, other factors, such as malocclusion, pain, or discomfort when related to dentofacial discrepancies cannot be discarded, as they might induce an elevation of the IL-1β levels in saliva. Recent evidence has suggested a potential role for IL-10 toward the homeostasis in animal models of depression, secondary to inflammation^33^. The salivary levels of the anti-inflammatory cytokine IL-10 were undetectable in this study for most patients in either of the groups, except for two patients with high levels of this cytokine (one from the control group and the other from the test group). Therefore, it might be concluded that dentofacial deformities are not correlated to an IL-10 elevation. The discrepant data that was observed in the two patients might be related to another medical condition that has not been the focus of this study. It is tempting to suggest that the IL-1β/IL-10 proportion might be reversed in long-term follow-up of the post-OS patients.

The stimulation of IDO-1 by the inflammatory cytokines in mood disorders leads to the activation of the tryptophan/kynurenine pathway, triggering the formation of the kynurenic and quinolinic acids, among other metabolites. Kynurenic acid has been suggested as a neuroprotective molecule, acting as an antagonist of the glutamate receptors, besides other receptors. Oppositely, quinolinic acid might activate the glutamate receptors, by displaying a neurotoxic effect^10,34^. Of note, kynurenic acid salivary contents have been correlated with psychological stress in schizophrenic individuals^35^. Taking into account the poor scores of OQLQ in the patients with dentofacial deformities, it was relevant to evaluate the levels of kynurenine metabolites by mass spectrometry. No significant differences in the salivary levels of kynurenine, kynurenic, or quinolinic acids between the control and test groups were observed. These results uphold the absence of significant differences as to the depression, anxiety, and stress levels, as indicated by the DASS-21 scale.

The test group participants showed higher salivary glutamate levels (+ 25%) than did the control subjects. In this group, the salivary glutamate levels were positively correlated with the OQLQ function domain scores but not with the other dimensions of this questionnaire. It is reasonable to propose that the glutamate levels might be related to the functional disorders in the patients with dentofacial deformities. Having said that, this proposition needs to be interpreted cautiously when viewing the nature of the correlation analysis and the need for further studies to better understand the pathophysiological role of glutamate in the patients undergoing OS. The patients seeking OS often display head and neck pain, as well as fatigue symptoms^36^, which might explain the elevated levels of glutamate, besides their relationship with the functional disabilities. Accordingly, salivary glutamate contents were found elevated in the female chronic migraineurs when compared to the individuals with episodic attacks, or in the controls^28^. Moreover, a recent study has revealed an increase of salivary glutamate contents in patients that were diagnosed with temporomandibular disorder-myalgia, despite a lack of correlation with the pain scores^37^. Opposite to that, Dawson et al. did not show any variation of the glutamate muscle levels in patients with temporomandibular disorders^38^. It is important to remark that the IL-1β or glutamate salivary levels did not show any significant correlation with age or BMI, which might be factors contributing to the variations of the inflammatory biomarkers in this research. Yet again, this evidence requires careful interpretation and further investigation.

## Strengths and limitations

The patients allocated in the control and test groups were matched by age and sex, and they presented very similar demographic and anthropometric features, revealing the homogeneity of the study sample. In addition, the patients allocated in either group were under orthodontic treatment, which is relevant considering the possible alterations of the salivary inflammatory mediators that are associated with the orthodontic devices and the driven movements. As a limitation of the present study, the sample size was small and represented only a population that was seeking treatment in the University dental clinics, with an evident intragroup age variation, which enrolled both adolescents and middle-aged individuals. In this report, the levels of the inflammatory biomarkers were assessed only in the stimulated saliva, which might produce different results when in comparison with the unstimulated saliva. The salivary flow rates were not measured, despite the regular amounts of saliva that were collected from most of the subjects. Finally, the use of additional instruments for evaluating the psychosocial aspects of the individuals seeking OS would be very helpful, as well as the investigation of other inflammatory mediators, permitting further advances for comprehending the psychobiological features of this type of population.

## Conclusion

The results have demonstrated that the patients undergoing OS presented higher salivary levels of IL-1β and glutamate. The study partially rejected the null hypothesis of no differences regarding the salivary levels of the inflammatory mediators when in comparison with the control individuals, as no variations of the kynurenine metabolites, IL-6, and IL-10 were identified. Altogether, the present data opens a new avenue for investigating the biological alterations in those patients that present dentofacial deformities.

## Methods

### Study design, participants and outcome measures

This case–control study was performed in ethical agreement with the Helsinki Declaration^39^ (ninth version, 2013; pre-trial registration number 12283019.7.0000.5336) and conformed to The STrengthening the Reporting of OBservational Studies in Epidemiology (STROBE) guidelines^40^. All of the procedures were approved by the Research Ethics Committee of the Pontifícia Universidade Católica do Rio Grande do Sul (CEP-PUCRS; report number 3.343.197), Brazil, on May 23, 2019. Informed consent was obtained from each patient, and they were assured of data confidentiality. When the individuals were under 18 years old, informed consent was obtained from a parent and/or legal guardian.

**Figure 4:**
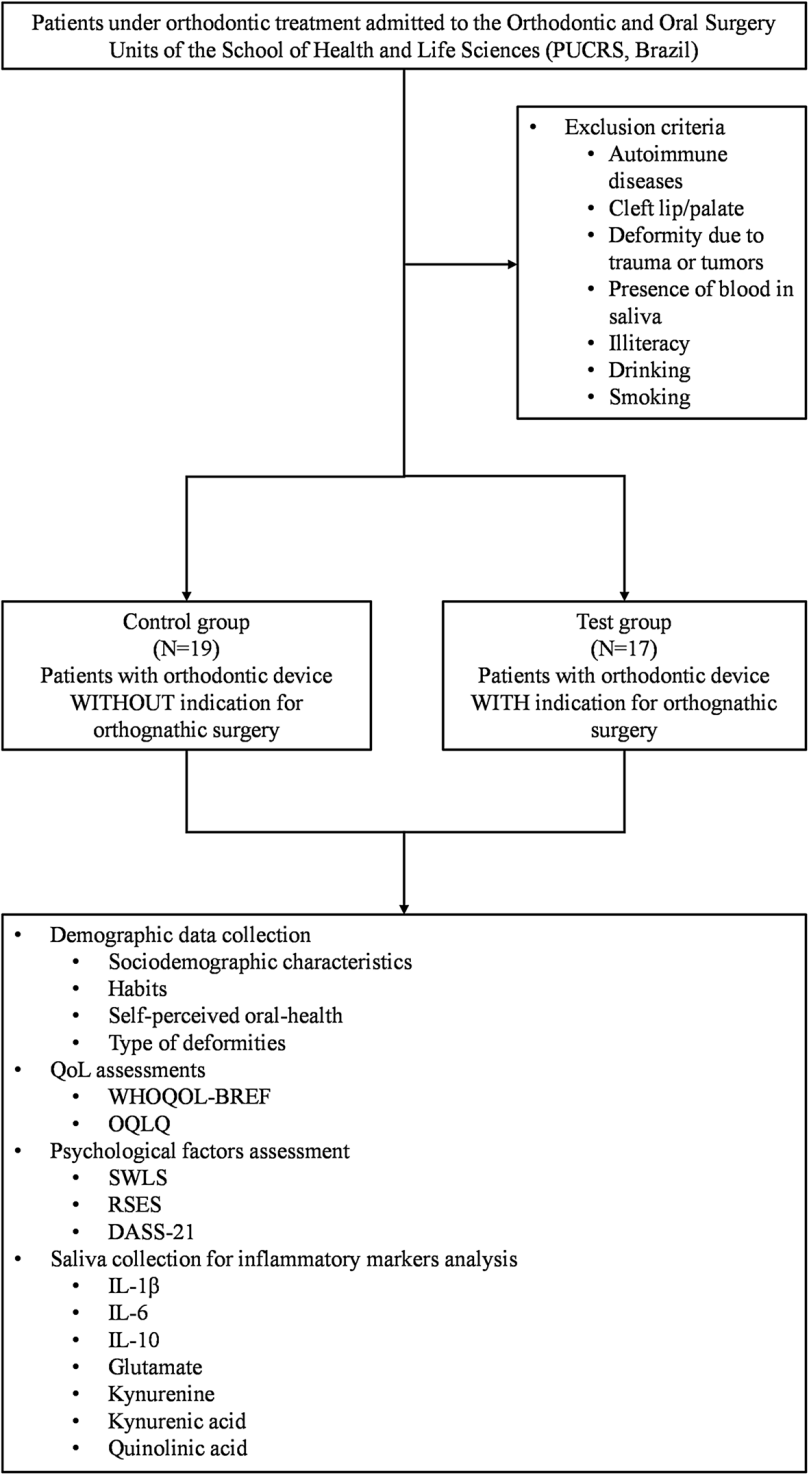
Flowchart of the Study Design. The patients undergoing orthodontic treatment that were admitted to the Orthodontic and Oral Surgery Units of the School of Health and Life Sciences (PUCRS, Brazil) were recruited for this study. The eligible patients were allocated into two different groups, according to the need for OS. Afterward, the participants answered sociodemographic, QoL, and psychological factors questionnaires. Saliva was collected for the analyses of the inflammatory markers.

The present study enrolled 36 patients, which were distributed into two groups: control (n = 19) and test (n = 17), matched by age and sex. The patients under an orthodontic treatment, without surgical indication, were included in the control group. The test group was composed of patients with skeletal deformities, whilst undergoing OS. The patients were recruited from the Orthodontic and Oral Surgery Units of the School of Health and Life Sciences (PUCRS, Brazil), from June to December 2019. Those patients with cleft lip/palate, deformity due to trauma or tumors, autoimmune diseases, and illiteracy were excluded from the study. Drinking alcohol and/or smoking, as well as the presence of blood in the saliva were also noted in the exclusion criteria. The flowchart, depicted in Fig. 4, displays the overall study design. The sociodemographic data, habits, anthropometric features (height in m, weight in g, and BMI), self-perceived oral health, sleep quality, and physical activity levels, besides the type of deformity of the participants, are provided in Table 1 and in Supplementary Table S1.

When reviewing the absence of previous studies, which have evaluated the salivary levels of the inflammatory biomarkers in patients requiring OS, the sample size was calculated with a minimum difference of 15% between the control and test groups, with 80% power, and 5% level of significance, hence providing a number of 18 participants per group (G*Power Version 3.1.9.7; Germany). The main outcomes were the salivary levels of the cytokines, glutamate, and kynurenine metabolites. The secondary outcomes were the questionnaires for the assessment of QoL and the psychological factors.

### Assessment of QoL

Two questionnaires were adopted to evaluate the QoL of the participants: the World Health Organization QoL Questionnaire BREF version (WHOQOL-BREF)^41^, and the Portuguese-Brazilian version of the Orthognathic QoL Questionnaire (OQLQ)^42^. The WHOQOL-BREF consisted of 26-items divided into the following domains: physical, psychological, social, and the environment. Questions 1 and 2 were analyzed individually, and they considered the overall QoL and the general health facet, respectively. The final scores varied from 0 to 100 points, with the higher scores indicating a better QoL. The OQLQ comprised of 22-items divided into four domains, such as the social aspects, dentofacial esthetics, function, and the awareness of dentofacial esthetics. The sum of the OQLQ domains ranged from 0 to 88 points, with higher scores indicating an inferior oral QoL.

### Psychological factors

The overall satisfaction levels were evaluated by using the Brazilian version of the Satisfaction with Life Scale (SWLS)^18^. This questionnaire was comprised of five questions with reference to the current satisfaction of life on a 7-point Likert scale. The higher scores indicated better levels (> 25) of life satisfaction. The RSES^20,43^ was applied to evaluate the self-esteem of the participants. The RSES consisted of 10 questions, ranging from 10 to 40, with ideal self-esteem scores varying from 15 to 25. The participants also completed the Depression, Anxiety, and Stress Scale (DASS-21), as previously described by Vignola and Tucci^44^. This questionnaire had 21 questions, divided into seven items, for each of the evaluated dimensions: depression, anxiety, and stress symptoms, which might vary from normal to extremely severe. The patients presenting high scores in any of the evaluated dimensions of DASS-21 were referred to the Psychology Research and Evaluation Service of the School of Health and Life Sciences (PUCRS, Brazil).

### Saliva collection

Stimulated whole saliva was collected from 9 to 11 a.m., according to the methodology previously described^29,30^. The patients were fasting for at least one hour before the saliva collection. Following a mouth rinse with water, the patients were instructed to rest in a seated position and chew orthodontic rubber bands for 1 min. The saliva was collected for 5 min in 5-ml sterile flasks and immediately stored at − 80 °C until analysis. On the day prior to the experiments, the samples were centrifuged at 3000 rpm for 10 min at 4 °C, and the supernatant was used for the analyses, as described below.

### Determination of cytokines

The salivary levels of interleukin-1β (IL-1β), interleukin-6 (IL-6), and interleukin-10 (IL-10) were determined by the sandwich enzyme-linked immunosorbent assay (ELISA) when using DuoSet commercial kits, according to the fabricant instructions. The directions recommended saliva centrifugation and the use of the aqueous layer for the assay (R&D Systems; Minneapolis, MN, USA). The results were expressed in picograms per ml. The kits and the limits of detection were: human IL-1β/IL-1F2 (DY201-05; 3.91 to 250 pg/ml); human IL-6 (DY206-05; 9.38 to 600 pg/ml), and human IL-10 (DY217B-05; 31.2 to 2.000 pg/ml). The experiments were run in duplicate.

### Determination of glutamate and the kynurenine metabolites

The levels of glutamate and the kynurenine pathway-related components (kynurenine, kynurenic acid, and quinolinic acid) were measured by LC–MS/MS, as described by Michael et al.^45,46^, with some adaptations. Briefly, 500-μl of saliva was mixed with 100 μl of ammonium acetate (25 mM; pH 9.0). Subsequently, the samples were centrifuged at 14,000 rpm for 5 min at 4 °C^45,46^. The supernatant was injected into the ACQUITY UPLC I-Class System that was coupled to the Xevo TQ-S micro (Waters; Milford, MA, USA), and the samples were run in duplicate. The chromatographic separations were performed by using a Zorbax^®^ Eclipse Plus C18 RRHD column (Agilent; Paolo Alto, CA, USA), featuring a 2.1-mm inner diameter, 50-mm length, and 1.8-µm particle size. The flow rates were 2.5 mM ammonium acetate (eluent A); acetonitrile with 2.5 mM of ammonium acetate (eluent B); and the mobile phase was 0.2 ml/min, with a column temperature of 40 °C. A gradient was used, starting at 10% of eluent B, subsequently increasing to 90% in 4 min, while remaining at this condition for one min. The initial condition was recovered and kept for 7.5 min. Five microliters of the samples were injected into the UHPLC System. The monitored transitions (m/z) were: glutamate (148 > 84), kynurenine (209.2 > 94), kynurenic acid (190 > 89), and quinolinic acid (168 > 78). The results were expressed as ng/ml for glutamate and pg/ml for the other metabolites. The quantifications were performed by external standardization. The calibration curves were obtained with the following concentrations, accompanied by R-squared (R^2^), and the standard deviation of the residuals (Sy.x): glutamate (50, 250, 500, 750, and 1000 ng/mL; R^2^ = 0.9924; Sy.x = 38.36); kynurenine (0.05, 0.25, 0.5, and 0.75 ng/mL; R^2^ = 0.9904; Sy.x = 0.04328); kynurenic acid (0.005, 0.025, 0.05, 0.075, and 0.1 ng/mL; R^2^ = 0.9901; Sy.x = 0.004395), and quinolinic acid (0.05, 0.25, 0.5, and 0.75 ng/mL; R^2^ = 0.9976; Sy.x = 0.02165).

### Statistical analysis

The data was expressed as mean ± SD. The data normality was checked by the Kolmogorov–Smirnov test. The statistical analyses were performed by the Unpaired Student’s t-test, or Two-Way ANOVA, followed by Sidak’s multiple comparisons test. For the correlation assessments, Pearson’s correlation coefficient was used. The frequency data was analyzed by using the Chi-square test for trend. *P*-values less than 0.05 were considered significant. All of the statistical tests and graphs were performed when using GraphPad Prism version 8.4.3 for Windows (GraphPad Software; San Diego, California, USA).

## Supplementary Information

Supplementary Information.
