# Supplementary material for: Psychological symptoms and salivary inflammatory biomarkers in patients with dentofacial deformities: a case–control study
Source: Sci Rep. 2021 May 26;11:11083. doi: 10.1038/s41598-021-90721-6 (PMC8155030; doi:10.1038/s41598-021-90721-6)
Supplement: Supplementary file 1 — Supplementary Information. [file 41598_2021_90721_MOESM1_ESM.docx]

**SUPPLEMENTARY DATA**

**Psychological symptoms and salivary inflammatory biomarkers in patients with dentofacial deformities: a case-control study**

Maria C.C. Volkweis^1,3^, Gabriela W. Neculqueo^1,3^, Raquel D.S. Freitas^3^, Ana P.A. Dagnino^2,3^, Guilherme G. Fritscher^4^, Tatiana Q. Irigaray^5^, *Maria M. Campos^1,2,3^

^1^Programa de Pós-graduação em Odontologia, Escola de Ciências da Saúde e da Vida, Pontifícia Universidade Católica do Rio Grande do Sul, Porto Alegre, RS, Brazil.

^2^Programa de Pós-graduação em Medicina e Ciências da Saúde, Escola de Medicina, Pontifícia Universidade Católica do Rio Grande do Sul, Porto Alegre, RS, Brazil.

^3^Centro de Pesquisa em Toxicologia e Farmacologia, Escola de Ciências da Saúde e da Vida, Pontifícia Universidade Católica do Rio Grande do Sul, Porto Alegre, RS, Brazil.

^4^Ambulatório de Cirurgia Oral, Escola de Ciências da Saúde e da Vida, Pontifícia Universidade Católica do Rio Grande do Sul, Porto Alegre, RS, Brazil.

^5^Programa de Pós-graduação em Psicologia, Escola de Ciências da Saúde e da Vida, Pontifícia Universidade Católica do Rio Grande do Sul, Porto Alegre, RS, Brazil.

***Corresponding author:** Maria M. Campos, Escola de Ciências da Saúde e da Vida, Pontifícia Universidade Católica do Rio Grande do Sul, Avenida Ipiranga, 6681, Partenon, Porto Alegre, RS 90619-900, Brazil. Tel: +55 51 33320 3677.

Email address: [maria.campos@pucrs.br](mailto:maria.campos@pucrs.br) [camposmmartha@yahoo.com](mailto:camposmmartha@yahoo.com)

Maria C. C. Volkweis: 0000-0001-6199-9258

Maria M. Campos: 0000-0001-7738-9892

**Table S1.** Religion, occupation and domestic animals of participants.

|  | **Groups** | |  |
| --- | --- | --- | --- |
|  | **Control** | **Test** | ***p-*value** |
| **N** | 19 | 17 |  |
| **Religion** |  |  |  |
| Atheist or agnostic | 6 | 4 | 0.2879 |
| Catholic | 12 | 9 |  |
| Evangelical | - | 3 |  |
| Other religions | 1 | 1 |  |
| **Occupation** |  |  |  |
| Dentist | 3 | - | 0.5676 |
| Engineer | - | 1 |  |
| Housekeeping | 1 | 1 |  |
| Policeman | - | 1 |  |
| Physical therapist | - | 1 |  |
| Secretary | - | 2 |  |
| Student | 12 | 6 |  |
| Others | 3 | 4 |  |
| Without occupation | - | 1 |  |
| **Domestic animals** |  |  |  |
| Dog | 7 | 9 | 0.6305 |
| Cat | 2 | 1 |  |
| Dog and cat | 4 | 1 |  |
| Birds | - | 1 |  |
| Horse | - | 1 |  |
| No animals | 6 | 4 |  |

*n*, participants in each group. Chi-square test for trend.

**Table S2.** Frequency of severity ratings according to DASS-21 scale.

|  | **Groups** | | | | | | | | | |  |
| --- | --- | --- | --- | --- | --- | --- | --- | --- | --- | --- | --- |
|  | **Control** | | | | | **Test** | | | | | **Adjusted *p*-value** |
| **N** | 19 | | | | | 17 | | | | |  |
| **Domain** |  | | | | |  | | | | |  |
|  | Normal | Mild | Moderate | Severe | X-severe | Normal | Mild | Moderate | Severe | X-severe |  |
|  |  |  |  |  |  |  |  |  |  |  |  |
| **Depression** | 13 (68%) | 1 (5%) | 1 (5%) | 2 (10%) | 2 (10%) | 11 (65%) | 4 (24%) | 1 (6%) | 1 (6%) | - | 0.3681 |
| **Anxiety** | 12 (63%) | - | 4 (21%) | - | 3 (16%) | 12 (71%) | - | 2 (12%) | - | 3 (18%) | 0.6365 |
| **Stress** | 10 (53%) | 1 (5%) | 5 (26%) | - | 3 (16%) | 12 (71%) | - | 3 (18%) | - | 2 (12%) | 0.4218 |

*n*, participants in each group; *X-severe*, extremely severe. Chi-square test for trend.
